# Improvement of beta-cell function in conjunction with glycemic control after medical nutrition therapy in newly-diagnosed type 2 diabetes mellitus

**DOI:** 10.1186/s12902-022-01064-w

**Published:** 2022-06-04

**Authors:** Mitsuyoshi Takahara, Toshihiko Shiraiwa, Yoshifumi Maeno, Kaoru Yamamoto, Yuka Shiraiwa, Yoko Yoshida, Norio Nishioka, Naoto Katakami, Iichiro Shimomura

**Affiliations:** 1 Department of Diabetes Care Medicine, Osaka University Graduate School of Medicine, 2-2 Yamadaoka, Suita, 565-0871 Japan; 2 Shiraiwa Medical Clinic, 4-10-24 Hozenji, Kashiwara City, Osaka 582-0005 Japan; 3 Department of Metabolic Medicine, Osaka University Graduate School of Medicine, 2-2 Yamadaoka, Suita, 565-0871 Japan

**Keywords:** Disposition index, Hemoglobin A1c, Medical nutrition therapy, Newly-diagnosed type 2 diabetes mellitus

## Abstract

**Background:**

The current study aimed to reveal the correlation of beta-cell function and insulin sensitivity with glycemic control and weight control before and after medical nutrition therapy (MNT) in patients with newly-diagnosed type 2 diabetes mellitus.

**Methods:**

We retrospectively analyzed consecutive 68 patients with newly-diagnosed type 2 diabetes mellitus who started MNT without antihyperglycemic medications and underwent a 75-g oral glucose tolerance test (OGTT) before and after the therapy. Beta-cell function was evaluated by the OGTT-derived disposition index, whereas insulin sensitivity was evaluated by Matsuda’s insulin sensitivity index.

**Results:**

After 4.0 ± 1.5 months of MNT, mean HbA1c and body mass index significantly decreased from 9.6 ± 1.8% to 7.2 ± 1.0% and from 26.9 ± 4.1 to 25.4 ± 3.7 kg/m^2^ (both *P* < 0.001), while the median disposition index and Matsuda’s index significantly increased from 0.34 (0.20–0.68) to 0.88 (0.53–1.52) (*P* < 0.001) and from 4.70 (2.95–5.93) to 5.17 (3.48–6.89) (*P* = 0.003), respectively. The disposition index was significantly correlated with HbA1c levels both before and after MNT (*r* = -0.61 and -0.68; both *P* < 0.001). The magnitude of the correlation after MNT was not different from that before MNT (*P* = 0.42). Matsuda’s index was correlated not with HbA1c levels but with body mass index, both before (*r* = 0.07 [*P* = 0.57] and *r* = -0.58 [*P* < 0.001]) and after MNT (*r* = -0.01 [*P* = 0.95] and *r* = -0.52 [*P* < 0.001]).

**Conclusions:**

Beta-cell function was improved in conjunction with glycemic control after MNT in patients with newly-diagnosed type 2 diabetes mellitus. Insulin sensitivity was linked with weight control rather than glycemic control.

**Supplementary Information:**

The online version contains supplementary material available at 10.1186/s12902-022-01064-w.

## Background

Type 2 diabetes mellitus is characterized by both insulin resistance and beta-cell dysfunction. Insulin resistance is closely linked with overweight and obesity, and weight reduction through medical nutrition therapy (MNT) will ameliorate insulin resistance, improving glucose metabolism [1]. On the other hand, the role of MNT in the amelioration of beta-cell dysfunction remains less clear. While beta-cell function is progressively impaired as a natural history of type 2 diabetes mellitus [2], sustained hyperglycemia will excessively deteriorate beta-cell function [3], and forced correction of hyperglycemia can ameliorate the deteriorated function [4]. In this sense, beta-cell function will not follow monotonous decrease but rather be variable to some extent. Our previous pilot study of 14 patients newly diagnosed with type 2 diabetes mellitus suggested that beta-cell function could be improved together with glycemic control, weight control, and insulin sensitivity after MNT, even without any aid of antihyperglycemic medications including exogenous insulin administration [5]. However, our previous study mainly focused on the change of beta-cell function after MNT in patients with considerably poor glycemic control; the study population was limited to patients with baseline hemoglobin A1c (HbA1c) levels of 8% (64 mmol/mol) or higher, and the correlation was only assessed between beta-cell function and glycemic control after MNT. The correlation was not compared with that before MNT (i.e., at baseline). The correlations of insulin sensitivity and weight control also remained unrevealed. Furthermore, limiting the study population to those with high HbA1c levels would cause a statistical problem known as range restriction during the correlation analysis.

Our present study aimed to reveal the correlation of beta-cell function and insulin sensitivity with glycemic control and weight control before and after MNT in patients with newly-diagnosed type 2 diabetes mellitus who started their treatment with MNT.

## Methods

### Study population

The current study retrospectively analyzed consecutive 68 patients with newly-diagnosed type 2 diabetes mellitus who started their treatment with MNT at Shiraiwa Medical Clinic, Kashiwara City, Osaka, Japan, between June 2016 and February 2020, and underwent a 75-g oral glucose tolerance test (OGTT) both before and after MNT. Patients who were already diagnosed with type 2 diabetes mellitus but remained untreated at another medical institution were also included in the current study, regardless of their duration from the diagnosis. Patients taking any antihyperglycemic medications were excluded. Between June 2016 and February 2020, a total of 147 patients with newly-diagnosed type 2 diabetes mellitus started their treatment at Shiraiwa Medical Clinic. Of the 147 patients, 23 patients started an antihyperglycemic agent within three months, and 11 patients were lost to follow-up during three months. The remaining 113 patients were treated with MNT without any antihyperglycemic medications for at least 3 months. Of the 113 patients, 68 patients underwent a 75-g OGTT both before and after MNT, and were included in the current study (Additional file 1: Table S1). All study patients underwent a 75-g OGTT at the first month, and started standard MNT, without introducing any antihyperglycemic medications. Patients were basically followed-up every month, and registered dietitians instructed and supported healthy eating habits with balanced foods. Excessive restriction of specific nutrients or calories were not encouraged. Details of MNT instruction by registered dietitians are summarized in Additional file 2: Appendix S1. After several months, usually after three to six months, a 75-g OGTT was re-performed to re-assess their glucose metabolism. Note that OGTTs were performed in clinical practice at the clinic; the data helped understand pathophysiology and plan the subsequent treatment strategies, and also helped show patients how effective their lifestyle modification was for the improvement of glucose metabolism. OGTTs were performed just in clinical practice, not primarily for study purpose. During a 75 g OGTT, blood samples were collected to measure glucose and insulin levels at 0, 30, 60, and 120 min.

All data used in the current study were retrospectively derived from medical records. The current study was in accordance with Declaration of Helsinki, and was approved by the ethics committees of Shiraiwa Medical Clinic (approval number, 2,020,902; approval date, September 2, 2020) and that of Osaka University Hospital (approval number 15395–3; approval date, September 7, 2020). Since the current study retrospectively used existing data, informed consent was exempted and instead relevant information regarding the study was open to the public, according to the Ethical Guidelines for Medical and Health Research Involving Human Subjects in Japan.

### Definitions

Body mass index (BMI) was calculated as body weight in kilograms divided by the square of height in meters. Family history of diabetes was determined when relatives within the second degree had diabetes. The information on family history of diabetes and duration of diabetes was based on self-report and medical records. Beta-cell function as well as insulin sensitivity was calculated from OGTT data as follows. Insulin sensitivity was assessed as Matsuda’s insulin sensitivity index [6]. Beta-cell function was evaluated with the disposition index, calculated as the product of ΔI_0–120_/ΔG_0–120_ (insulin secretion index) and Matsuda’s insulin sensitivity index [7–9]. The insulin secretion index (ΔI_0–120_/ΔG_0–120_) was calculated as the ratio of incremental area under the curve of insulin levels divided by the incremental area under the curve of glucose levels during a 120-min OGTT; incremental areas under the curve of glucose and insulin levels during the OGTT were calculated according the trapezoid rule [7–9]. We also calculated homeostasis model assessment of b-cell (HOMA-β) and that of insulin resistance (HOMA-IR) as alternative markers of insulin secretion capacity and insulin resistance (i.e., impaired insulin sensitivity), respectively [10].

### Statistical analysis

Data are presented as mean ± standard deviation or median (interquartile range) for continuous variables, and as frequency (proportion) for discrete variables, respectively, unless otherwise mentioned. A *P* value of < 0.05 was considered statistically significant, and 95% confidence intervals (CIs) are reported where appropriate. During parametric statistical analysis, the disposition index, Matsuda’s insulin sensitivity index, the insulin sensitivity index (ΔI_0–120_/ΔG_0–120_), HOMA-β, and HOMA-IR, all of which had a right-skewed distribution, were log-transformed. Baseline characteristics were compared between patients with baseline HbA1c levels < 9% (75 mmol/mol) and those with baseline HbA1c levels ≥ 9% (75 mmol/mol) by Welch’s *t* test for continuous variables and by the chi-squared test for discrete variables. The change of metabolic profiles after MNT was tested by the paired *t* test. The correlations of HbA1c levels and BMI with the disposition index, Matsuda’s index, the insulin sensitivity index (ΔI_0–120_/ΔG_0–120_), HOMA-β, and HOMA-IR before and after MNT were assessed using the Pearson’s method, and the difference between the correlations before MNT and the corresponding ones after MNT was tested by 5,000-time bootstrap resampling. We additionally performed the subgroup analysis based on baseline HbA1c levels (≥ 9% [75 mmol/mol] and < 9% [75 mmol/mol]). During the subgroup analysis, correlation coefficients were corrected for range restriction using the Thorndike’s formula. As supplementary exploratory analysis, we investigated whether clinical characteristics other than HbA1c levels and BMI would have any residual association with the disposition index, Matsuda’s index, the insulin sensitivity index (ΔI_0–120_/ΔG_0–120_), HOMA-β, and HOMA-IR. The investigation was conducted using the linear regression model adjusted for HbA1c levels and BMI. All statistical analyses were performed using R version 4.1.1 (R Development Core Team, Vienna, Austria).

## Results

**Fig. 1:**
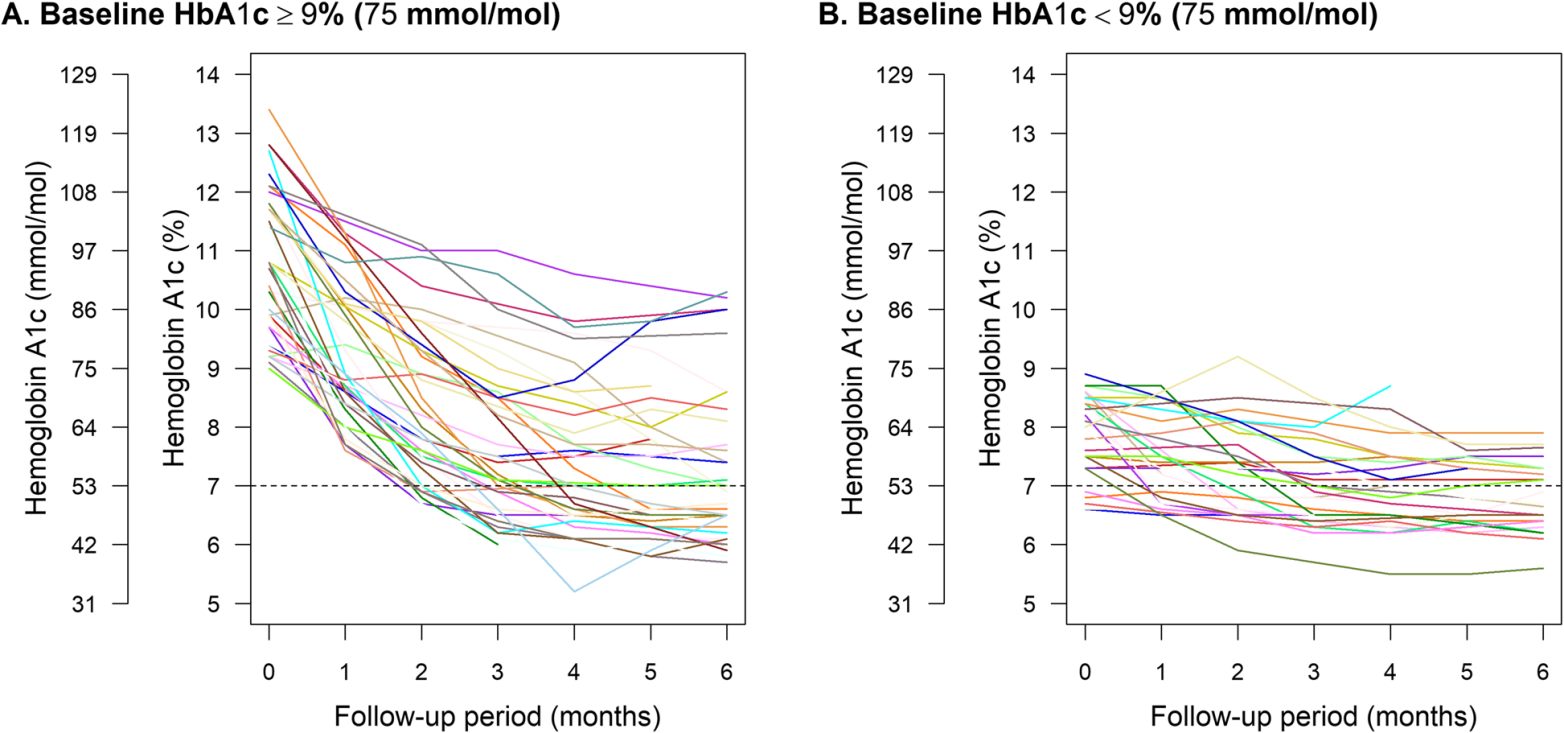
Change of HbA1c level over 6 months in patients with baseline HbA1c levels ≥ 9% (75 mmol/mol) (*n* = 39) (**A**) and those with lower baseline HbA1c levels (*n* = 29) (**B**). Data were HbA1c level under medical nutrition therapy without antihyperglycemic medications. In patients who started an antihyperglycemic medication within 6 months, only the data before the start were plotted

Clinical characteristics of the study population are summarized in Table 1. The mean age was 52 ± 10 years, mean baseline HbA1c level was 9.6 ± 1.8% (81 ± 20 mmol/mol), and mean BMI was 26.9 ± 4.1 kg/m^2^. Compared with patients with baseline HbA1c levels < 9% (75 mmol/mol), those with higher HbA1c levels had lower levels of the disposition index, the insulin secretion index (ΔI0-120/ΔG0-120), and HOMA-β, while other clinical characteristics including the Matsuda’s insulin sensitivity index and HOMA-IR were not significantly different (Table 1). The change of HbA1c levels after MNT is shown in Fig. 1. The proportion of achieving HbA1c < 7% (53 mmol/mol) without antidiabetic medications at 6 months was 51% (95% CI, 34% to 68%) in patients with baseline HbA1c levels ≥ 9% (75 mmol/mol) and 54% (95% CI, 34% to 72%) in those with lower baseline HbA1c levels. The second 75-g OGTT was performed at 4.0 ± 1.5 months after the start of MNT. The disposition index and Matsuda’s insulin sensitivity index significantly increased after MNT, while HbA1c levels and BMI significantly decreased (Table 2). The insulin sensitivity index (ΔI0-120/ΔG0-120) and HOMA-β significantly increased, while HOMA-IR significantly decreased (Additional file 1: Table S2).

**Table 1:** Baseline characteristics of study population

	Overall population (*n* = 68)	Baseline HbA1c < 9% (75 mmol/mol) (*n* = 29)	Baseline HbA1c ≥ 9% (75 mmol/mol) (*n* = 39)	*P* value
Age (years)	52 ± 10	53 ± 11	52 ± 9	0.92
Male sex	52 (76%)	21 (72%)	31 (79%)	0.70
Smoking				0.49
Never	24 (35%)	10 (34%)	14 (36%)	
Past	21 (31%)	7 (24%)	14 (36%)	
Current	23 (34%)	12 (41%)	11 (28%)	
Hypertension	26 (38%)	12 (41%)	14 (36%)	0.84
Dyslipidemia	46 (68%)	21 (72%)	25 (64%)	0.64
Duration of diabetes (years)	0 (0–1)	0 (0—1)	0 (0—2)	0.50
Family history of diabetes^a^	42 (68%)	19 (70%)	23 (66%)	0.91
eGFR (ml/min/1.73 m^2^)	91.6 ± 18.0	92.4 ± 21.1	90.4 ± 12.9	0.64
eGFR category				0.98
≥ 90 ml/min/1.73 m^2^	34 (50%)	20 (51%)	14 (48%)	
60–89 ml/min/1.73 m^2^	32 (47%)	17 (44%)	15 (52%)	
45–60 ml/min/1.73 m^2^	2 (3%)	2 (5%)	0 (0%)	
< 45 ml/min/1.73 m^2^	0 (0%)	0 (0%)	0 (0%)	
Albuminuria (mg/gCre)	7.2 (3.4–17.5)	7.6 (3.6–21.4)	6.7 (3.3–9.1)	0.38
Albuminuria category				1.00
Normoalbuminuria	58 (85%)	33 (85%)	25 (86%)	
Microalbuminuria	10 (15%)	6 (15%)	4 (14%)	
Macroalbuminuria	0 (0%)	0 (0%)	0 (0%)	
HbA1c (%)	9.6 ± 1.8	7.9 ± 0.7	10.8 ± 1.2	< 0.001
(mmol/mol)	81 ± 20	62 ± 8	95 ± 14	
Body mass index (kg/m^2^)	26.9 ± 4.1	27.0 ± 4.2	26.8 ± 4.0	0.89
Disposition index (unit)	0.34 (0.20–0.68)	0.62 (0.39–0.78)	0.25 (0.17–0.37)	< 0.001
Matsuda’s index (unit)	4.70 (2.95–5.93)	4.71 (2.42–6.18)	4.69 (3.25–5.82)	0.67
Insulin secretion index (unit)	0.08 (0.05–0.16)	0.05 (0.04–0.10)	0.12 (0.08–0.24)	< 0.001
HOMA-β (unit)	17.8 (10.7–24.6)	15.4 (9.2–21.8)	22.3 (14.7–36.8)	0.004
HOMA-IR (unit)	2.41 (1.80–4.51)	2.62 (2.07–3.86)	2.18 (1.60–4.70)	0.52

Data are means ± standard deviations, medians (interquartile ranges), or frequencies (percentages).

*eGFR* estimated Glomerular Filtration Rate, *HbA1c* Hemoglobin A1c, *HOMA-β* Homeostasis Model Assessment of b-cell, *HOMA-IR* Homeostasis Model Assessment of Insulin Resistance.

^a^Data on family history of diabetes were missing in 6 patients. Matsuda’s index denotes Matsuda’s insulin sensitivity index. The insulin secretion index denotes ΔI_0-120_/ΔG_0-120_.

**Table 2:** Change after medical nutrition therapy

	At baseline	After MNT	*P* value
Overall population (*n* = 68)			
HbA1c (%)	9.6 ± 1.8	7.2 ± 1.0	< 0.001
(mmol/mol)	81 ± 20	55 ± 11	
Body mass index (kg/m^2^)	26.9 ± 4.1	25.4 ± 3.7	< 0.001
Disposition index (unit)	0.34 (0.20–0.68)	0.88 (0.53–1.52)	< 0.001
Matsuda’s insulin sensitivity index (unit)	4.70 (2.95–5.93)	5.17 (3.48–6.89)	0.003
Baseline HbA1c ≥ 9% (75 mmol/mol) (*n* = 39)			
HbA1c (%)	10.8 ± 1.2	7.5 ± 1.1	< 0.001
(mmol/mol)	95 ± 14	58 ± 12	
Body mass index (kg/m^2^)	26.8 ± 4.0	25.3 ± 3.3	< 0.001
Disposition index (unit)	0.25 (0.17–0.37)	0.87 (0.48–1.48)	< 0.001
Matsuda’s insulin sensitivity index (unit)	4.69 (3.25–5.82)	5.35 (3.85–7.17)	0.031
Baseline HbA1c < 9% (75 mmol/mol) (*n* = 29)			
HbA1c (%)	7.9 ± 0.7	6.9 ± 0.7	< 0.001
(mmol/mol)	62 ± 8	52 ± 7	
Body mass index (kg/m^2^)	27.0 ± 4.2	25.6 ± 4.1	< 0.001
Disposition index (unit)	0.62 (0.39–0.78)	1.00 (0.69–1.51)	< 0.001
Matsuda’s insulin sensitivity index (unit)	4.71 (2.42–6.18)	4.66 (2.75–6.47)	0.034

Data are mean ± standard deviations or medians (interquartile ranges)

*MNT* Medical Nutrition Therapy

**Fig. 2:**
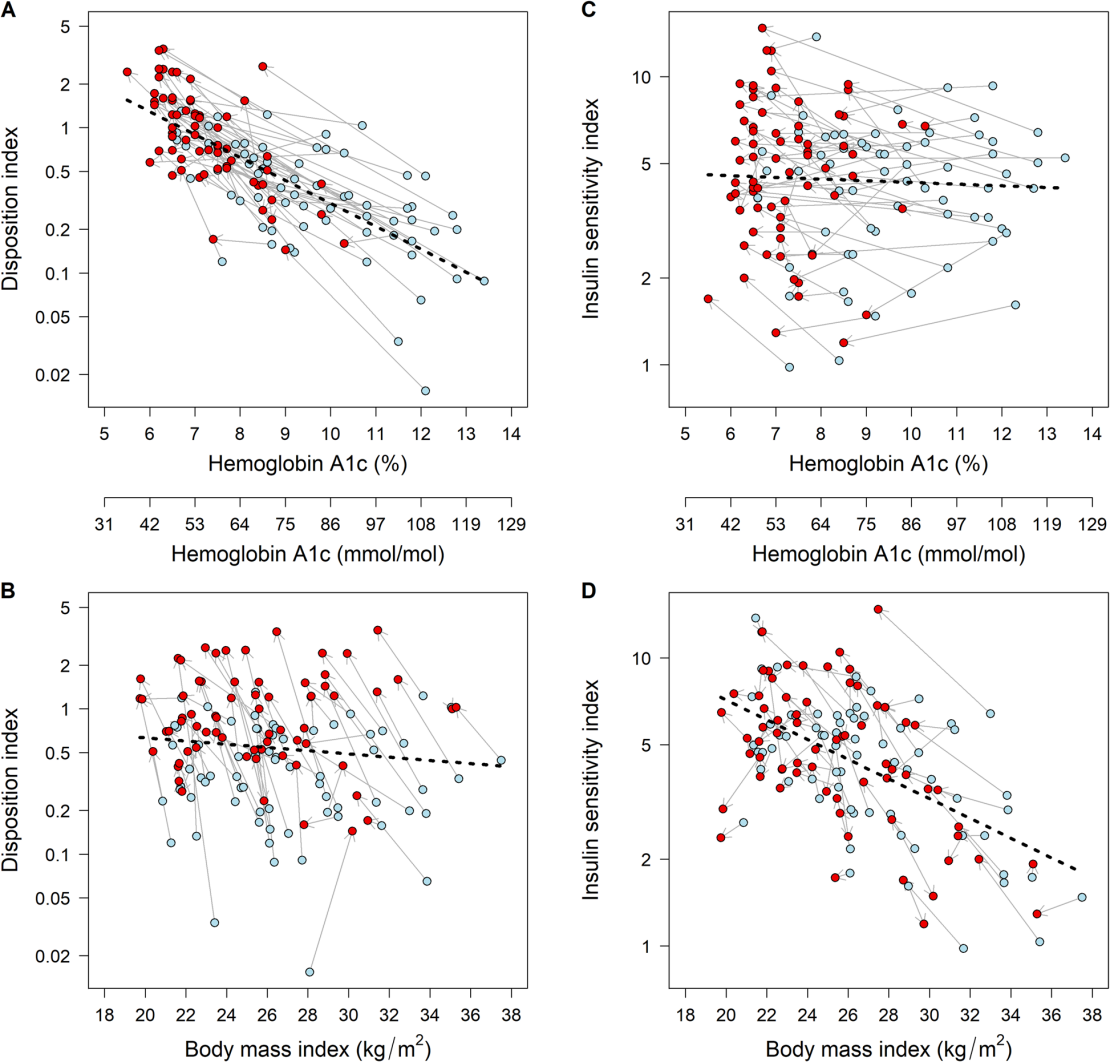
Correlation of hemoglobin A1c and body mass index with disposition index and Matsuda’s insulin sensitivity index (*n* = 68). Light blue dots represent values measured at baseline, whereas red dots represent those measured after medical nutrition therapy. Grey arrows show the change in individual subjects

Table 3 demonstrates the correlations of HbA1c levels and BMI with the disposition index and Matsuda’s insulin sensitivity index. HbA1c levels at the corresponding time point were significantly inversely correlated with the disposition index both before MNT (i.e., at baseline) and after MNT (*r* = -0.61 [*P* < 0.001] at baseline and -0.68 [*P* < 0.001] after MNT), but were not significantly correlated with Matsuda’s insulin sensitivity index (*r* = 0.07 [*P* = 0.57] at baseline and -0.01 [*P* = 0.95] after MNT). On the other hand, BMI was significantly inversely associated with Matsuda’s insulin sensitivity index (*r* = -0.58 [*P* < 0.001] at baseline and -0.52 [*P* < 0.001] after MNT), but was not significantly associated with the disposition index (*r* = -0.02 [*P* = 0.90] at baseline and *r* = -0.02 [*P* = 0.90] after MNT). The correlations of HbA1c levels and BMI with the disposition index and Matsuda’s insulin sensitivity index after MNT were not significantly different from those at baseline (all *P* > 0.05) (the rightmost column in Table 3). The correlations are illustrated in Fig. 2. The correlations of HbA1c levels and BMI with the insulin sensitivity index (ΔI_0–120_/ΔG_0–120_), HOMA-β, and HOMA-IR are shown in Additional file 1: Table S3 and Figure S1. HbA1c levels and BMI were significantly associated with the insulin sensitivity index (ΔI_0–120_/ΔG_0–120_) and HOMA-β, while BMI, but not HbA1c levels, was significantly associated with HOMA-IR, both at baseline and after MNT. Again, correlations after MNT were not significantly different from the corresponding ones at baseline. As demonstrated in Additional file 1: Tables S4 and S5, those correlations were not different between patients with baseline HbA1c levels ≥ 9% (75 mmol/mol) and those with lower baseline HbA1c levels. No clinical features other than HbA1c levels or BMI had significant residual association with the disposition index, Matsuda’s index, the insulin sensitivity index (ΔI_0–120_/ΔG_0–120_), HOMA-β, or HOMA-IR (Additional file 1: Tables S6).

**Table 3:** Correlation of HbA1c and BMI with disposition index and Matsuda’s insulin sensitivity index (*n* = 68)

	At baseline (A)	After medical nutrition therapy (B)	Difference between A and B
HbA1c and disposition index	-0.61 [-0.74 to -0.44] (*P* < 0.001)	-0.68 [-0.79 to -0.53] (*P* < 0.001)	-0.07 [-0.22 to 0.11] (*P* = 0.42)
HbA1c and Matsuda’s insulin sensitivity index	0.07 [-0.17 to 0.30] (*P* = 0.57)	-0.01 [-0.25 to 0.23] (*P* = 0.95)	-0.08 [-0.30 to 0.15] (*P* = 0.49)
BMI and disposition index	-0.02 [-0.25 to 0.22] (*P* = 0.90)	-0.02 [-0.25 to 0.22] (*P* = 0.90)	0.00 [-0.24 to 0.25] (*P* = 0.99)
BMI and Matsuda’s insulin sensitivity index	-0.58 [-0.72 to -0.40] (*P* < 0.001)	-0.52 [-0.68 to -0.32] (*P* < 0.001)	0.06 [-0.07 to 0.19] (*P* = 0.35)

Data are correlation coefficients [95% confidence intervals] (*P* values). BMI and HbA1c levels were those measured at the same time point as the disposition index and Matsuda’s insulin sensitivity index.

*BMI* Body Mass Index, *HbA1c* Hemoglobin A1c

## Discussion

The current study demonstrated that the disposition index significantly increased after MNT, and was considerably correlated with decreased HbA1c levels both before and after MNT, in patients with newly-diagnosed type 2 diabetes mellitus. The correlation after MNT was not different from that before MNT. The disposition index increased after MNT in conjunction with HbA1c reduction. On the other hand, Matsuda’s insulin sensitivity index was linked with BMI.

Previous studies demonstrated that beta-cell function was cross-sectionally correlated with glycemic control in patients with type 2 diabetes mellitus [11, 12]. However, it remained unrevealed whether the correlation was similarly observed once a glucose-lowering intervention improved glycemic control. We revealed that the correlation observed before MNT was still preserved after MNT, wherein glycemic control was drastically changed, and that beta-cell function substantially changed in conjunction with HbA1c change. The progressive nature of beta-cell dysfunction in type 2 diabetes mellitus is well recognized [2], and it has been often outlined that antihyperglycemic medications will be ultimately required as beta-cell function is progressively deteriorated [13–15]. However, the current finding suggests that HbA1c goals could be achieved by MNT without medications even if beta-cell function was severely impaired before MNT. Beta-cell function might be more variable than expected.

The variability of beta-cell function could be explained in the context of glucotoxicity. Beta-cell function will be impaired by sustained exposure to hyperglycemia (so called as glucotoxicity) [3], and the impairment can be ameliorated by the correction of hyperglycemia [16]. Beta-cell function is a key determinant of glycemic control, and at the same time is potentially affected by glycemic control. The two are closely linked with each other. In the management of type 2 diabetes mellitus, the restoration of beta-cell function often attracts clinical attention [17], and its easy and simple evaluation in clinical practice has been discussed [18]. The current finding that HbA1c levels were considerably correlated with the disposition index regardless of the completion of MNT suggests that HbA1c levels per se would roughly show beta-cell function at the moment in patients without antihyperglycemic medications.

Insulin sensitivity, assessed with Matsuda’s insulin sensitivity index, was not so strongly correlated with glycemic control, but was rather considerably linked with weight control. It would be reasonable that the results of HOMA-IR, an index of insulin resistance (or impaired insulin sensitivity), were a mirror of those of Matsuda’s insulin sensitivity index. Although both insulin resistance and beta-cell dysfunction are key pathophysiologic features of type 2 diabetes mellitus and will be improved after MNT, the involvement in the improvement of glycemic control and weight control after MNT would be different between the two.

The ΔI_0-120_/ΔG_0-120_ and HOMA-β were inversely correlated with HbA1c levels and positively correlated with BMI, which would be because the parameters represent the insulin secretion capacity, which is not only regulated by beta-cell function but also compensates for insulin resistance (or impaired insulin sensitivity) [7–9, 19].

The current study has several limitations. First, beta-cell function was assessed with the disposition index calculated as a product of the insulin secretion index (ΔI_0-120_/ΔG_0-120_) and the Matsuda’s insulin sensitivity index. Conceptually, the validity of the disposition index, calculated as a product of insulin secretion and sensitivity indices, is derived from a hyperbolic relationship between the two indices. Although the product of the ΔI_0-120_/ΔG_0-120_ and Matsuda’s index has been adopted as a reliable marker in many clinical studies conducted overseas [7–9], it remained unrevealed whether the validity is similarly guaranteed in a Japanese population. Future studies using other assessments of beta-cell function will be needed to validate the current findings. Second, the sample size of the current study was small. The minimum correlation coefficient *r* that could be detected with the power of ≥ 80% was calculated to be 0.33. Furthermore, the current sample size was insufficient to develop the full regression model (using all potential confounders) or statistical information-based variable selection model to assess whether HbA1c levels and BMI would be associated with the disposition index and Matsuda’s insulin sensitivity index independently of all candidates for covariates. Whether the correlations would be explained by other confounders remained unrevealed in the current study, and future studies are needed to prove their independent associations. In addition, although the current supplementary exploratory analysis showed that no clinical characteristics other than HbA1c levels or BMI had a significant residual association with the disposition index or Matsuda’s insulin sensitivity index, the non-significance might come from the small sample size. Future studies are needed to validate those findings. Third, OGTTs were performed in clinical practice, not for study purpose, and therefore not all patients of interest underwent an OGTT twice. In clinical settings, the second OGTT was more likely to be performed in patients whose glucose metabolism was expected to be considerably changed, whereas it was less likely to be performed in patients whose glucose metabolism was not expected to be so changed. This retrospective study included only patients who underwent an OGTT twice, which would cause a possible selection bias in the current study. Fourth, detailed data were not available on patient characteristics, including lifestyles, eating habits, and physical activity, which might be potential factors associated with the improvement of beta-cell function and the achievement of glycemic goals.

## Conclusions

The disposition index was considerably correlated with HbA1c levels, both before and after MNT, in patients with newly-diagnosed type 2 diabetes mellitus. On the other hand, Matsuda’s insulin sensitivity index was correlated with BMI. The magnitude of the correlation after MNT was not different from that before MNT. The disposition index increased after MNT in conjunction with HbA1c reduction, while Matsuda’s insulin sensitivity index increased in conjunction with BMI reduction.

## Supplementary Information

**Additional file 1:** **Table S1.** Patients who were newly diagnosed with type 2 diabetes and started their treatment at the study clinic during the study period. **Table S2.** Change of other insulin secretion/sensitivity-related indices after MNT. **Table S3.** Correlation of HbA1c and BMI with other insulin secretion/sensitivity-related indices (*n* = 68). **Figure S1.** Correlation of HbA1c and BMI with insulin sensitivity index (ΔI0–120/ΔG0–120), HOMA-β, and HOMA-IR (*n* = 68). **Table S4.** Correlation of HbA1c and BMI with disposition index and Matsuda’s insulin sensitivity index by baseline HbA1c (*n* = 68). **Table S5.** Correlation of HbA1c and BMI with other insulin secretion/sensitivity-related indices by baseline HbA1c (*n* = 68). **Table S6.** Association of clinical characteristics with disposition index, Matsuda’s insulin sensitivity index, and other indices (*n* = 68). 

**Additional file 2:** **Appendix S1.** Medical nutrition therapy at Shiraiwa Medical Clinic. 

## Data Availability

The datasets generated and analysed during the current study are not publicly available due to ethical reason, but available from the corresponding author on reasonable request and with permission of the ethics committees of Shiraiwa Medical Clinic and Osaka University Hospital.

## Abbreviations

BMI: Body mass index
eGFR: Estimated glomerular filtration rate
HbA1c: Hemoglobin A1c
HOMA-β: Homeostasis model assessment of b-cell
HOMA-IR: Homeostasis model assessment of insulin resistance
MNT: Medical nutrition therapy
OGTT: Oral glucose tolerance test
