# Supplementary material for: Improvement of beta-cell function in conjunction with glycemic control after medical nutrition therapy in newly-diagnosed type 2 diabetes mellitus
Source: BMC Endocr Disord. 2022 Jun 4;22:150. doi: 10.1186/s12902-022-01064-w (PMC9167542; doi:10.1186/s12902-022-01064-w)
Supplement: Supplementary file 1 — Additional file 1: Table S1. Patients who were newly diagnosed with type 2 diabetes and started their treatment at the study clinic during the study period. Table S2. Change of other insulin secretion/sensitivity-related indices after MNT. Table S3. Correlation of HbA1c and BMI with other insulin secretion/sensitivity-related indices (n = 68). Figure S1. Correlation of HbA1c and BMI with insulin sensitivity index (ΔI0–120/ΔG0–120), HOMA-β, and HOMA-IR (n = 68). Table S4. Correlation of HbA1c and BMI with disposition index and Matsuda’s insulin sensitivity index by baseline HbA1c (n = 68). Table S5. Correlation of HbA1c and BMI with other insulin secretion/sensitivity-related indices by baseline HbA1c (n = 68). Table S6. Association of clinical characteristics with disposition index, Matsuda’s insulin sensitivity index, and other indices (n = 68). [file 12902_2022_1064_MOESM1_ESM.pdf]

Additional file 1: Table S1. Patients who were newly diagnosed with type 2 diabetes and started their treatment at the study clinic during the study period

	Baseline HbA1c ≥9.0% (≥75 mmol/mol)	Baseline HbA1c 7.0–8.9% (53–74 mmol/mol)	Baseline HbA1c <7.0% (<53 mmol/mol)	Total
Treated with MNT without any antidiabetic agent for more than 3 mo	43 (39)	36 (24)	34 (5)	113 (68)
HbA1c < 7% (53 mmol/mol) without any antidiabetic agent at 6 mo	19 (19)	17 (10)	31 (5)	67 (34)
HbA1c ≥ 7% (53 mmol/mol) or antidiabetic agent use at 6 mo	22 (18)	16 (13)	1 (0)	39 (31)
Data missing on HbA1c at 6 mo	2 (2)	3 (1)	2 (0)	7 (3)
Starting an antidiabetic agent within 3 mo	6	13	4	23
Lost to follow-up within 3 mo	4	6	1	11
Total	53	55	39	147

Data are presented as the number of patients. Numbers in parentheses indicate the number of patients who were treated with MNT without any antidiabetic agent for more than 3 months and underwent a 75-g OGTT before and after MNT (i.e., patients who were included in the current study). HbA1c, hemoglobin A1c; MNT, medical nutrition therapy; OGTT, oral glucose tolerance test.

Additional file 1: Table S2. Change of other insulin secretion/sensitivity-related indices after MNT

	At baseline	After MNT	P value
Overall population (n = 68)			
Insulin sensitivity index ($\Delta I_{0\text{-}120}/\Delta G_{0\text{-}120}$)	0.08 (0.05–0.16)	0.16 (0.10–0.36)	<0.001
HOMA-β	17.8 (10.7–24.6)	25.0 (15.3–47.2)	<0.001
HOMA-IR	2.41 (1.80–4.51)	1.84 (1.21–2.86)	<0.001
Baseline HbA1c ≥9% (75 mmol/mol) (n = 39)			
Insulin sensitivity index ($\Delta I_{0\text{-}120}/\Delta G_{0\text{-}120}$)	0.05 (0.04–0.10)	0.14 (0.10–0.36)	<0.001
HOMA-β	15.4 (9.2–21.8)	23.0 (14.8–44.4)	<0.001
HOMA-IR	2.62 (2.07–3.86)	1.84 (1.19–2.39)	<0.001
Baseline HbA1c <9% (75 mmol/mol) (n = 29)			
Insulin sensitivity index ($\Delta I_{0\text{-}120}/\Delta G_{0\text{-}120}$)	0.12 (0.08–0.24)	0.20 (0.12–0.39)	<0.001
HOMA-β	22.3 (14.7–36.8)	28.06 (18.2–49.3)	0.009
HOMA-IR	2.18 (1.60–4.70)	1.84 (1.23–3.38)	0.003

Data are medians (interquartile ranges). HbA1c, hemoglobin A1c; HOMA-β, homeostasis model assessment of b-cell. HOMA-IR, homeostasis model assessment of insulin resistance; MNT, medical nutrition therapy.

Additional file 1: Table S3. Correlation of HbA1c and BMI with other insulin secretion/sensitivity-related indices (n = 68)

	Correlation at baseline (A)	Correlation after MNT (B)	Difference between A and B
HbA1c and insulin secretion index ($\Delta I_{0\text{-}120}/\Delta G_{0\text{-}120}$)	-0.56 [-0.70 to -0.37] (P<0.001)	-0.55 [-0.70 to -0.36] (P<0.001)	0.01 [-0.17 to 0.21] (P=0.89)
HbA1c and HOMA-β	-0.42 [-0.60 to -0.20] (P<0.001)	-0.45 [-0.62 to -0.24] (P<0.001)	-0.04 [-0.22 to 0.19] (P=0.76)
HbA1c and HOMA-IR	0.16 [-0.08 to 0.38] (P=0.19)	0.22 [-0.02 to 0.44] (P=0.071)	0.06 [-0.17 to 0.27] (P=0.60)
BMI and insulin secretion index ($\Delta I_{0\text{-}120}/\Delta G_{0\text{-}120}$)	0.29 [0.06 to 0.50] (P=0.015)	0.31 [0.08 to 0.51] (P=0.009)	0.02 [-0.15 to 0.18] (P=0.81)
BMI and HOMA-β	0.48 [0.28 to 0.65] (P<0.001)	0.47 [0.26 to 0.64] (P<0.001)	-0.01 [-0.18 to 0.18] (P=0.90)
BMI and HOMA-IR	0.58 [0.39 to 0.72] (P<0.001)	0.54 [0.35 to 0.69] (P<0.001)	-0.03 [-0.19 to 0.12] (P=0.67)

Data are correlation coefficients [95% confidence intervals] (P values). BMI, body mass index; HbA1c, hemoglobin A1c; HOMA-β, homeostasis model assessment of b-cell. HOMA-IR, homeostasis model assessment of insulin resistance; MNT, medical nutrition therapy.

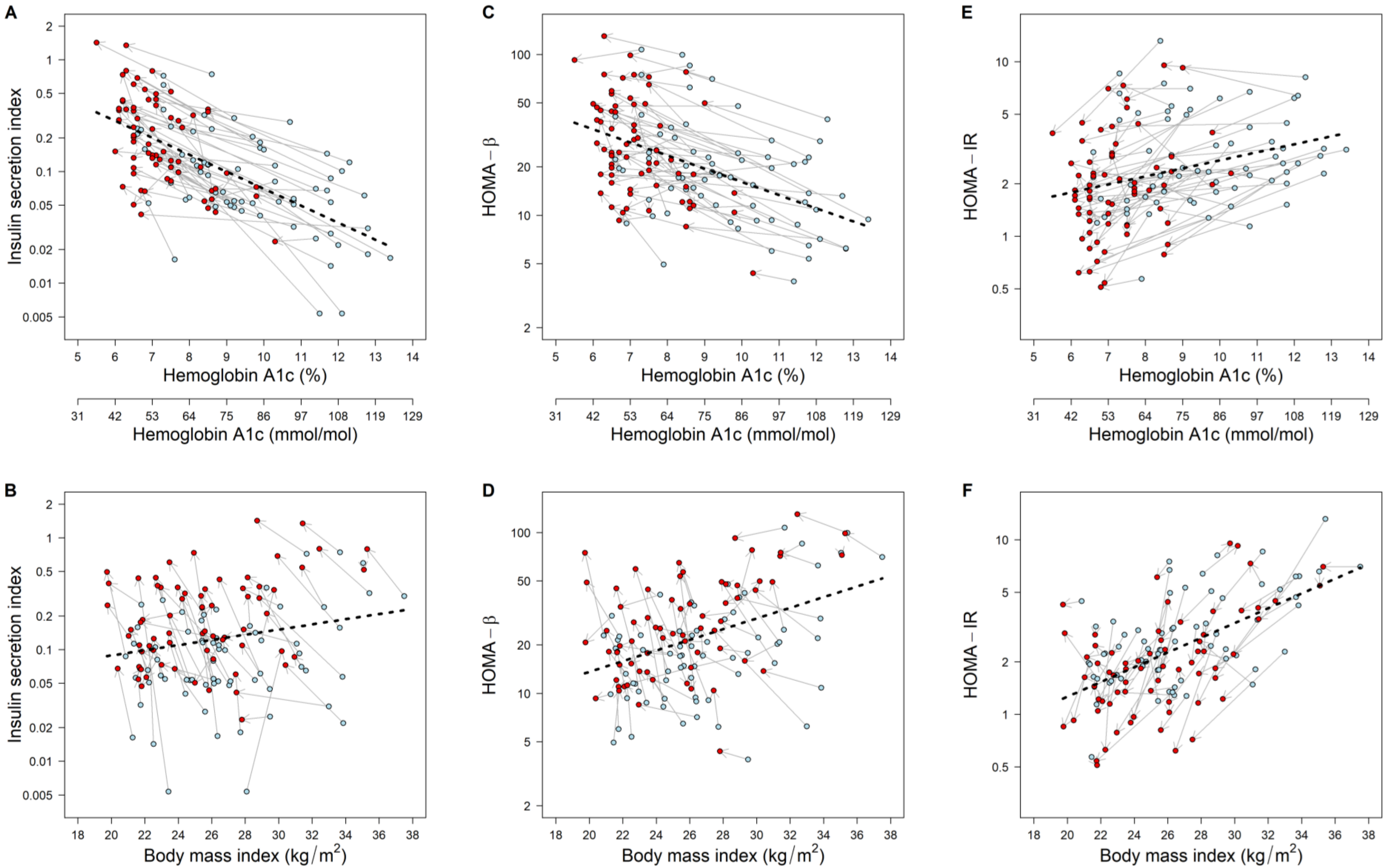

Additional file 1: Figure S1. Correlation of HbA1c and BMI with insulin sensitivity index ($\Delta I_{0-120}/\Delta G_{0-120}$), HOMA-β, and HOMA-IR (n = 68)

Light blue and red dots represent values measured at baseline and after medical nutrition therapy, respectively. Grey arrows show the change in individual subjects. BMI, body mass index; HbA1c, hemoglobin A1c; HOMA-β, homeostasis model assessment of b-cell. HOMA-IR, homeostasis model assessment of insulin resistance.

Additional file 1: Table S4. Correlation of HbA1c and BMI with disposition index and Matsuda's insulin sensitivity index by baseline HbA1c (n = 68)

	Correlation at baseline (A)		Correlation after MNT (B)		Difference between A and B	
HbA1c and disposition index		P=0.47		P=0.18		P=0.15
Baseline HbA1c ≥9% (75 mmol/mol)	-0.59 [-0.77 to -0.32]		-0.78 [-0.93 to -0.31]		-0.19 [-0.50 to 0.31]	
Baseline HbA1c <9% (75 mmol/mol)	-0.75 [-0.90 to -0.50]		-0.65 [-0.89 to 0.02]		0.09 [-0.26 to 0.79]	
HbA1c and Matsuda's insulin sensitivity index		P=0.99		P=0.35		P=0.81
Baseline HbA1c ≥9% (75 mmol/mol)	0.06 [-0.43 to 0.46]		-0.30 [-0.78 to 0.46]		-0.35 [-1.00 to 0.54]	
Baseline HbA1c <9% (75 mmol/mol)	-0.09 [-0.43 to 0.24]		-0.23 [-0.63 to 0.37]		-0.14 [-0.66 to 0.56]	
BMI and disposition index		P=0.49		P=0.89		P=0.76
Baseline HbA1c ≥9% (75 mmol/mol)	-0.10 [-0.44 to 0.23]		-0.16 [-0.65 to 0.57]		-0.06 [-0.66 to 0.76]	
Baseline HbA1c <9% (75 mmol/mol)	-0.11 [-0.44 to 0.21]		-0.08 [-0.55 to 0.54]		0.03 [-0.52 to 0.75]	
BMI and Matsuda's insulin sensitivity index		P=0.31		P=0.61		P=0.17
Baseline HbA1c ≥9% (75 mmol/mol)	-0.43 [-0.69 to -0.05]		-0.75 [-0.89 to -0.46]		-0.32 [-0.72 to 0.08]	
Baseline HbA1c <9% (75 mmol/mol)	-0.41 [-0.69 to 0.05]		-0.69 [-0.87 to -0.37]		-0.29 [-0.75 to 0.13]	

Data are correlation coefficients corrected for range restriction [95% confidence intervals]. P values are for difference between the subgroup with baseline HbA1c ≥9% (75 mmol/mol) (n = 39) and that with baseline HbA1c <9% (75 mmol/mol) (n = 29). BMI, body mass index; HbA1c, hemoglobin A1c; MNT, medical nutrition therapy.

Additional file 1: Table S5. Correlation of HbA1c and BMI with other insulin secretion/sensitivity-related indices by baseline HbA1c (n = 68)

	Correlation at baseline (A)	Correlation after MNT (B)	Difference between A and B
HbA1c and insulin secretion index ($\Delta I_{0-120}/\Delta G_{0-120}$)	P=0.72	P=0.51	P=0.91
Baseline HbA1c ≥9% (75 mmol/mol)	-0.57 [-0.76 to -0.27]	-0.51 [-0.87 to 0.32]	0.06 [-0.40 to 0.91]
Baseline HbA1c <9% (75 mmol/mol)	-0.61 [-0.79 to -0.34]	-0.36 [-0.81 to 0.31]	0.25 [-0.29 to 0.93]
HbA1c and HOMA-β	P=0.90	P=0.40	P=0.97
Baseline HbA1c ≥9% (75 mmol/mol)	-0.51 [-0.74 to -0.13]	0.05 [-0.65 to 0.71]	0.57 [-0.24 to 1.27]
Baseline HbA1c <9% (75 mmol/mol)	-0.50 [-0.74 to -0.12]	-0.07 [-0.63 to 0.42]	0.43 [-0.24 to 0.99]
HbA1c and HOMA-IR	P=0.39	P=0.83	P=0.10
Baseline HbA1c ≥9% (75 mmol/mol)	0.21 [-0.23 to 0.60]	0.52 [-0.20 to 0.84]	0.31 [-0.50 to 0.87]
Baseline HbA1c <9% (75 mmol/mol)	0.32 [-0.01 to 0.58]	0.37 [-0.15 to 0.67]	0.05 [-0.55 to 0.48]
BMI and insulin secretion index ($\Delta I_{0-120}/\Delta G_{0-120}$)	P=0.20	P=0.23	P=0.61
Baseline HbA1c ≥9% (75 mmol/mol)	0.10 [-0.32 to 0.46]	0.36 [-0.18 to 0.81]	0.26 [-0.38 to 0.92]
Baseline HbA1c <9% (75 mmol/mol)	0.16 [-0.25 to 0.51]	0.40 [-0.13 to 0.78]	0.24 [-0.36 to 0.81]
BMI and HOMA-β	P=0.11	P=0.19	P=0.83
Baseline HbA1c ≥9% (75 mmol/mol)	0.28 [-0.16 to 0.63]	0.70 [0.30 to 0.88]	0.42 [-0.10 to 0.89]
Baseline HbA1c <9% (75 mmol/mol)	0.34 [-0.07 to 0.69]	0.62 [0.20 to 0.85]	0.28 [-0.26 to 0.75]
BMI and HOMA-IR	P=0.18	P=0.15	P=0.88
Baseline HbA1c ≥9% (75 mmol/mol)	0.45 [0.14 to 0.69]	0.74 [0.37 to 0.90]	0.29 [-0.15 to 0.63]
Baseline HbA1c <9% (75 mmol/mol)	0.43 [0.02 to 0.68]	0.73 [0.40 to 0.90]	0.30 [-0.11 to 0.73]

Data are correlation coefficients corrected for range restriction [95% confidence intervals]. P values are for difference between the subgroup with baseline HbA1c ≥9% (75 mmol/mol) (n = 39) and that with baseline HbA1c <9% (75 mmol/mol) (n = 29). BMI, body mass index; HbA1c, hemoglobin A1c; HOMA-β, homeostasis model assessment of b-cell. HOMA-IR, homeostasis model assessment of insulin resistance; MNT, medical nutrition therapy.

Additional file 1: Table S6. Association of clinical characteristics with disposition index, Matsuda's insulin sensitivity index, and other indices (n = 68)

Dependent variable	Disposition index at baseline		Matsuda's index at baseline		Insulin secretion index at baseline		HOMA-β at baseline		HOMA-IR at baseline	
Adjustment	Unadjusted	Adjusted*	Unadjusted	Adjusted*	Unadjusted	Adjusted*	Unadjusted	Adjusted*	Unadjusted	Adjusted*
Age	0.10 (P=0.41)	0.11 (P=0.34)	0.35 (P=0.003)	0.06 (P=0.59)	-0.10 (P=0.41)	0.06 (P=0.59)	-0.31 (P=0.009)	-0.09 (P=0.42)	-0.38 (P=0.001)	-0.10 (P=0.40)
Male sex	-0.17 (P=0.16)	-0.13 (P=0.20)	-0.08 (P=0.54)	0.01 (P=0.90)	-0.11 (P=0.39)	-0.12 (P=0.23)	0.08 (P=0.49)	0.04 (P=0.69)	0.13 (P=0.27)	0.03 (P=0.77)
Smoking	0.10 (P=0.41)	-0.00 (P=0.97)	-0.02 (P=0.88)	0.01 (P=0.89)	0.10 (P=0.44)	-0.01 (P=0.91)	-0.00 (P=1.00)	-0.09 (P=0.34)	-0.04 (P=0.76)	-0.03 (P=0.75)
Hypertension	0.09 (P=0.48)	0.05 (P=0.60)	-0.03 (P=0.80)	0.01 (P=0.96)	0.09 (P=0.46)	0.04 (P=0.67)	-0.11 (P=0.38)	-0.16 (P=0.092)	-0.02 (P=0.86)	-0.04 (P=0.66)
Dyslipidemia	0.02 (P=0.86)	-0.01 (P=0.95)	-0.13 (P=0.30)	-0.14 (P=0.17)	0.09 (P=0.48)	0.07 (P=0.48)	0.07 (P=0.59)	0.06 (P=0.54)	0.08 (P=0.51)	0.10 (P=0.30)
eGFR	-0.07 (P=0.57)	0.01 (P=0.94)	-0.15 (P=0.21)	-0.14 (P=0.18)	0.02 (P=0.86)	0.08 (P=0.42)	0.06 (P=0.60)	0.10 (P=0.33)	0.20 (P=0.10)	0.15 (P=0.12)
Albuminuria	-0.01 (P=0.93)	0.01 (P=0.91)	-0.17 (P=0.16)	-0.00 (P=0.98)	0.08 (P=0.51)	0.01 (P=0.91)	0.18 (P=0.15)	0.05 (P=0.62)	0.10 (P=0.40)	-0.08 (P=0.47)
Duration of diabetes	-0.07 (P=0.59)	-0.07 (P=0.51)	-0.02 (P=0.85)	-0.13 (P=0.19)	-0.04 (P=0.72)	0.01 (P=0.88)	-0.09 (P=0.47)	0.00 (P=0.99)	0.07 (P=0.58)	0.18 (P=0.080)
Family history of diabetes†	0.17 (P=0.21)	0.08 (P=0.43)	0.01 (P=0.97)	0.09 (P=0.38)	0.14 (P=0.29)	0.02 (P=0.83)	0.05 (P=0.67)	-0.06 (P=0.53)	-0.02 (P=0.86)	-0.07 (P=0.49)

Data are standardized regression coefficients (p values). *Adjusted for HbA1c levels and body mass index which were measured at the same time point that each dependent variable was evaluated. Matsuda's index denotes Matsuda's insulin sensitivity index. The insulin secretion index denotes $\Delta I_{0-120}/\Delta G_{0-120}$. †Data on family history of diabetes were missing in 6 patients. eGFR, estimated glomerular filtration rate; HbA1c, hemoglobin A1c; HOMA-β, homeostasis model assessment of b-cell; HOMA-IR, homeostasis model assessment of insulin resistance.

Additional file 1: Table S6. Association of clinical characteristics with disposition index, Matsuda's insulin sensitivity index, and other indices (n = 68) (continued)

Dependent variable	Disposition index after MNT		Matsuda's index after MNT		Insulin secretion index after MNT		HOMA-β after MNT		HOMA-IR after MNT	
Adjustment	Unadjusted	Adjusted*	Unadjusted	Adjusted*	Unadjusted	Adjusted*	Unadjusted	Adjusted*	Unadjusted	Adjusted*
Age	-0.14 (P=0.24)	0.04 (P=0.74)	0.24 (P=0.045)	0.00 (P=0.98)	-0.27 (P=0.026)	0.03 (P=0.81)	-0.34 (P=0.004)	-0.01 (P=0.95)	-0.22 (P=0.065)	-0.04 (P=0.76)
Male sex	0.09 (P=0.45)	-0.00 (P=0.97)	-0.09 (P=0.46)	0.01 (P=0.95)	0.13 (P=0.28)	-0.01 (P=0.94)	0.12 (P=0.33)	-0.04 (P=0.70)	0.06 (P=0.65)	-0.01 (P=0.89)
Smoking	-0.04 (P=0.72)	-0.12 (P=0.18)	-0.04 (P=0.75)	-0.03 (P=0.81)	-0.01 (P=0.93)	-0.08 (P=0.39)	0.00 (P=1.00)	-0.07 (P=0.49)	0.06 (P=0.62)	0.07 (P=0.49)
Hypertension	-0.03 (P=0.81)	0.06 (P=0.49)	0.06 (P=0.60)	0.08 (P=0.44)	-0.06 (P=0.60)	-0.00 (P=0.99)	-0.08 (P=0.52)	-0.04 (P=0.71)	-0.06 (P=0.63)	-0.11 (P=0.29)
Dyslipidemia	0.01 (P=0.92)	0.07 (P=0.43)	-0.04 (P=0.74)	-0.04 (P=0.72)	0.04 (P=0.77)	0.08 (P=0.40)	-0.06 (P=0.65)	-0.02 (P=0.84)	0.02 (P=0.89)	-0.01 (P=0.96)
eGFR	-0.12 (P=0.33)	-0.05 (P=0.56)	-0.21 (P=0.080)	-0.18 (P=0.092)	0.04 (P=0.77)	0.07 (P=0.48)	0.09 (P=0.46)	0.10 (P=0.27)	0.22 (P=0.072)	0.16 (P=0.11)
Albuminuria	-0.02 (P=0.89)	-0.10 (P=0.29)	-0.27 (P=0.026)	-0.11 (P=0.32)	0.16 (P=0.20)	-0.01 (P=0.91)	0.25 (P=0.040)	0.05 (P=0.62)	0.21 (P=0.083)	0.07 (P=0.55)
Duration of diabetes	-0.24 (P=0.048)	-0.06 (P=0.56)	0.12 (P=0.34)	0.04 (P=0.71)	-0.27 (P=0.027)	-0.07 (P=0.48)	-0.22 (P=0.072)	-0.02 (P=0.83)	-0.05 (P=0.69)	-0.03 (P=0.80)
Family history of diabetes†	0.02 (P=0.90)	0.11 (P=0.29)	0.01 (P=0.93)	0.10 (P=0.37)	0.01 (P=0.96)	0.03 (P=0.81)	0.02 (P=0.87)	0.01 (P=0.96)	0.07 (P=0.58)	-0.05 (P=0.65)

Data are standardized regression coefficients (p values). *Adjusted for HbA1c levels and body mass index which were measured at the same time point that each dependent variable was evaluated. Matsuda's index denotes Matsuda's insulin sensitivity index. The insulin secretion index denotes $\Delta I_{0-120}/\Delta G_{0-120}$. †Data on family history of diabetes were missing in 6 patients. eGFR, estimated glomerular filtration rate; HbA1c, hemoglobin A1c; HOMA-β, homeostasis model assessment of b-cell; HOMA-IR, homeostasis model assessment of insulin resistance.
