# Supplementary material for: Improvement of beta-cell function in conjunction with glycemic control after medical nutrition therapy in newly-diagnosed type 2 diabetes mellitus
Source: BMC Endocr Disord. 2022 Jun 4;22:150. doi: 10.1186/s12902-022-01064-w (PMC9167542; doi:10.1186/s12902-022-01064-w)
Supplement: Supplementary file 2 — Additional file 2: Appendix S1. Medical nutrition therapy at Shiraiwa Medical Clinic. [file 12902_2022_1064_MOESM2_ESM.pdf]

Additional file 2: Appendix S1. Medical nutrition therapy at Shiraiwa Medical Clinic

Medical nutrition therapy (MNT) instructed at Shiraiwa Medical Clinic is broadly in line with the domestic recommendations by the Japan Diabetes Society (Japanese Clinical Practice Guideline for Diabetes 2019. Diabetology International 11:165–2, 2020). Healthy eating habits with balanced foods are supported, while excessive restriction of specific nutrients or calories are not encouraged. Fifteen registered dietitians are involved in MNT education at Shiraiwa Medical Clinic. Attending registered dietitians provides individual patients with instruction of MNT, based on the following four key components.

1) Collection of relevant information

To provide personalized effective MNT education, attending registered dietitians collect relevant information (see the panel below).

- Before providing MNT instruction, attending registered dietitians collect relevant information which is shared by nurses and doctors, and grasp patients' backgrounds and characteristics. Since dietary habits are often deeply linked with social backgrounds, information to collect is not limited to clinical aspects but include social aspects. Patients' main complaints are also checked at every clinic visit. Understanding patients' main complaints will help grasp what patients' attention centers on.
- Attending registered dietitians join patients' consultations with doctors at their first visit, and if appropriate at subsequent visits, to share doctors' explanations on medical assessments and plans to patients and patients' responses.
- Since important information on dietary behaviors can be obtained from small talk, attending registered dietitians pay attention to their communications with patients that apparently seem irrelevant to diets.
- Patients are asked to record their diets and to bring the records at their clinic visit. The records can be either written forms or photos. Information on dietary behaviors is collected from those records and additional verbal interviews. Other physical and laboratory data, including records of body weight and glucose monitoring, are used for retrospective assessment and discussion of dietary behaviors with patients. Physical activities are also checked, to assess their energy balance and way to spare time in their daily lives.
- Attending registered dietitians always read patients' emotion in their remarks, response, and facial expressions during the instruction and communication.

Panel. Information relevant to dietary behaviors

Information	Example	Means
Medical background	Physical and laboratory examinations, etiologies and pathogeneses of diabetes, comorbidity, complications, treatment plan, medication	Collection of information shared by doctors and nurses, attendance at patients' consultations with doctors, check of medical records
Social background	Lifestyle, environment, job, family, character, values, habits, hobbies, socioeconomic status	Medical interview, short talk with patients at clinic
Daily life	Diets: details of meals, snack, eating time, frequency of eating out, persons who cook<br>Physical activity: mobility, type and amount of physical activities, daily exercise	Record of diets, verbal interview, physical and laboratory data (including records of body weight and glucose monitoring)
Emotion	Notions of treatment, reluctance and willingness to modify dietary habits, refusal and approval	Remarks, responses, and facial expressions during instruction and communication with patients

2) Clarification and grasp of the point at issue

Based on the collected relevant information, attending registered dietitians seize the point at issue, from the following three viewpoints.

- Medical assessment: Patients are classified into the following three levels: (1) having medical problems that need to be solved (hyperglycemia, hypoglycemia, obesity, leanness, sarcopenia, etc.), (2) currently achieving provisional medical goals but being expected to achieve higher goals, and (3) being expected to keep current conditions.
- Lifestyle assessment: Patients are classified into the following three levels: (1) having problems that need to be solved (nutritional balance [both nutrients and total energy], dietary behaviors [including snack, alcohol consumption, and meal skips], physical activities, medication adherence, etc.), (2) having no serious problems and being expected to keep current conditions, and (3) living a "healthy" life, at the cost of their quality of life. When there are more than one problem that needs to be solved, attending registered dietitians weigh priorities.
- Barrier assessment: Patients are classified into the following two levels: (1) having barriers against lifestyle modification (cost, job [night shift, etc.], impaired cognition, living alone, lack of social and family support, etc.)

3) Assessment of the stage of behavior change

Since effective instructions vary with the stage of behavior change, attending registered dietitians recognize at which of the stages patients are (pre-contemplation, contemplation, preparation, action, or maintenance). Information collected on patients' emotion will help the recognition.

4) Instructive approach

The practical approach for MNT instruction is composed of five key actions: (1) obtaining patients' confidence, (2) sharing numerical goals, (3) setting behavioral goals, (4) supporting patients psychologically, and (5) encouraging patients' self-monitoring.

- Obtaining patients' confidence: Since patients' confidence are essential for effective MNT instruction, attending registered dietitians place first priority on obtaining their confidence, without forcing MNT on them, when patients offer resistance to intervention. Attending registered dietitians prompt patients to talk as freely and as much as possible and listen to them, especially at their first consulting. Instructions are provided by the same attending registered dietitians at every visit. During instructions, attending registered dietitians respect patients' beliefs and express a sympathetic response to patients' notions, even when patients' dietary behaviors based on their notions per se seemed questionable. Attending registered dietitians also attempt to express praise to patients, or admit patients' efforts, for at least one point (even minor one) at every instruction. At a visit with no MNT instruction scheduled, attending registered dietitians hold brief talk with patients during their waiting time, to keep regular contact with the patients.
- Sharing numerical goals: Attending registered dietitians share not only behavioral goals but also numerical goals with patients. Numerical goals include those of clinical parameters such as laboratory data and body weight. Setting numerical goals are often more effective for keeping patients' motivation than simply listing up what to do for lifestyle modification. When the numerical goals proposed by patients are unrealistically high, a short-term, realistic goal is set through discussion with patients. Attending registered dietitians help patients understand the importance of the numerical goals, sometimes explaining pathogeneses of diseases when appropriate.

- Setting behavioral goals: Attending registered dietitians make a full discussion with patients about behavioral goals for achieving the numerical goals. Attending registered dietitians do not force patients into medically ideal behaviors, but encourage patients to set behavioral goals by themselves. For the encouragement, a deep, practical discussion with patients is important. Although behavioral goals are set based on the numerical goals, it is always kept in mind that the ultimate goal is to promote their self-management and to maintain their daily life and quality of life at a level comparable to those in healthy individuals. After behavioral goals are determined, attending registered dietitians give patients relevant knowledge that will help them to achieve the behavioral goals. The knowledge includes that about the importance of MNT (association with pathogeneses and complications, effectiveness of lifestyle modification on health and disease, etc.), an overview of MNT (relationship between nutrients and metabolic control, energy balance, positioning of snack and alcohol, etc.), and practical strategies of MNT (ingredients, cooking methods, food plating techniques, etc.). Attending registered dietitians select which knowledge to give; the knowledge should be that patients can acquire and utilize, which will vary with the patient background. Instruction tools are sometimes used during the verbal instruction when appropriate. At some stages of behavior change, psychological supports have a priority over the determination of specific behavioral goals.
- Supporting patients psychologically: Attending registered dietitians always check whether patients' effort to modify their dietary behaviors are tremendous and causes psychological stresses. Since concerns and worries, even irrelevant to dietary habits (e.g., those about their family and job), will affect dietary behaviors, attending registered dietitians always try to recognize their concerns and worries, and support them psychologically. In addition, attending registered dietitians share patients' psychological changes with doctors and nurses, since such changes will also affect overall treatment strategies.
- Encouraging patients' self-monitoring: Attending registered dietitians encourage patients to record daily diets and body weight. Such records will also promote self-management of diseases. When patients also perform self-monitoring of blood glucose and blood pressure, their link is also supported. Attending registered dietitians give written feedback on their records, by writing down messages, comments, and goals on the records, since the written feedback will help patients maintain their motivation to improve their dietary behaviors.

Attending registered dietitians collect and update patients' relevant information at every visit. Clarification and grasp of the point at issue, assessment of the stage of behavior, and instructive approach are based on the updated information. Since patients' dietary behaviors are an important part of their daily life and are sometimes deeply linked with their beliefs and values, some patients are reluctant to be "instructed" in their dietary behaviors. Attending registered dietitians attempt to offer "supports" rather than "instructions" to them. Family members, especially those who cook daily meals, are encouraged to join consultations. Attending registered dietitians share the status of patients' MNT implementation and relevant problems, as well as patients' thoughts, with attending doctors and other staffs, to enable multifaceted supports of patients. Nutritional challenges and barrios are also discussed with other registered dietitians when appropriate. Shiraiwa Medical Clinic also post or display nutritional instruction tools (often relevant to individual seasons) at waiting rooms to make effective use of patients' waiting time.
